# Challenges of Diagnosing Antibody-Mediated Rejection: The Role of Invasive and Non-Invasive Biomarkers

**DOI:** 10.3390/medicina57050439

**Published:** 2021-05-03

**Authors:** Sambhavi Krishnamoorthy, Yousuf Kyeso

**Affiliations:** Division of Nephrology, The University of Chicago Medical Center, Chicago, IL 60637, USA

**Keywords:** kidney transplantation, antibody-mediated rejection, biomarkers

## Abstract

Kidney transplantation is the best treatment modality for end-stage kidney disease, leading to improvement in a patient’s quality and quantity of life. With significant improvements in short-term outcomes, prolonging long-term allograft and patient survival remain ongoing challenges. The ability to monitor allograft function, immune tolerance and predict rejection accurately would enable personalization and better prognostication during post-transplant care. Though kidney biopsy remains the backbone of transplant diagnostics, emerging biomarkers can help detecting kidney allograft injury early enough to prevent permanent damage and detect injury before it is clinically apparent. In this review, we summarize the recent biomarkers that have shown promise in the prediction of acute rejection with a focus on antibody-mediated rejection in kidney transplantation.

## 1. Introduction

In the current era of kidney transplantation, one-year patient and allograft survival exceed 95% [1]. Though a comparison of older cohorts of kidney transplant recipients to recent cohorts shows improving long-term outcomes, five-year patient and allograft survival continue to lag behind [2]. Advances in immunosuppression strategies, that have led to a decline in the incidence of acute rejection in the early postoperative period, are a major factor behind the remarkable short-term outcomes. However, antibody-mediated rejection (ABMR) continues to be a crucial factor in poor long-term graft survival and could present many years after transplantation [3,4,5].

The presence of pre-existing or de novo donor-specific antibodies (DSA), histologic evidence of glomerular injury and microcirculatory inflammation that have been implicated in development of transplant glomerulopathy and/or late kidney allograft failure are antibody-mediated processes [6,7,8,9,10]. Loupy et al. described the continuum of antibody-mediated damage, varying from an indolent process to functionally significant ABMR, leading to varying degrees of acute and chronic damage and eventual graft loss [11,12,13,14].

Though we are making major advances in understanding the pathophysiology and immunology of ABMR, we continue to rely on traditional markers like creatinine, proteinuria and kidney biopsy for screening and definitive diagnosis. Kidney biopsy, which is the gold standard to diagnose and differentiate between types of rejection and pathologic processes, is invasive, can have complications and is costly. Kidney biopsy is also subject to sampling error and interobserver variability between pathologists and different transplant centers [15]. Markers such as creatinine and DSAs may not be elevated until the disease process has reached an advanced stage and miss the window of treating an early subclinical rejection, which can impact long-term graft outcome [16].

**Figure 1:**
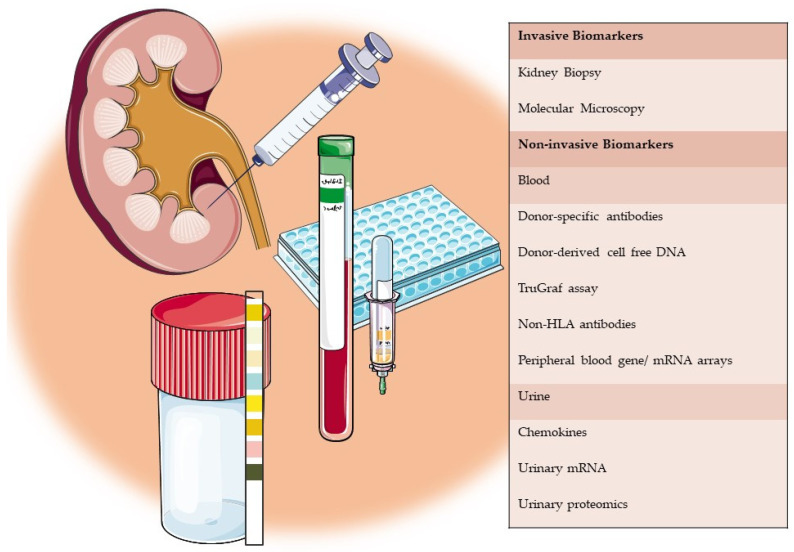
Overview of invasive and non-invasive biomarkers reviewed in this article.

Prior reviews have comprehensively described how the expansion of the “omics” field has led to a new era of transplant biomarkers which could be predictive, diagnostic or prognostic [17,18]. Biomarkers depending on the source could be invasive, as in requiring a tissue sample of the allograft, or be non-invasively obtained from blood or urine samples [19]. We review the current literature on potential biomarkers in the setting of ABMR from a diagnostic perspective with an emphasis on donor-derived cell-free DNA (dd-cfDNA) and molecular microscopy techniques (Figure 1).

## 2. Invasive Biomarkers

### 2.1. Kidney Biopsy—Banff Classification

Histologic evaluation via a kidney biopsy remains the gold standard of differentiating T cell-mediated rejection (TCMR) and ABMR. In 1993, the Banff schema was introduced with numeric coding of histologic features to allow for objectivity and definition of grades of acute rejection [20]. ABMR is further defined in the Banff 97 schema, as glomerulitis and peritubular capillaritis in the presence of donor-specific antibody or positive crossmatch [21]. Since then, the diagnosis of ABMR has evolved to acknowledge the recognition of C4d negative ABMR and the significance of non-HLA antibodies in the absence of donor-specific antibodies. The 2017 Banff meeting introduced the molecular analysis of biopsy tissue into the diagnostic algorithm with increased expression of gene transcripts or classifiers as evidence for the interaction of antibody with the allograft endothelium even in the absence of DSA or C4d positivity, as long as it is validated at the transplant center [22].

### 2.2. Molecular Microscopy Diagnostic System

The “molecular microscope diagnostic system” (MMDx) is a microarray-based system that examines messenger RNA (mRNA) expression patterns in transplant biopsy tissue to predict the diagnosis of acute TCMR or ABMR [23]. Halloran et al. and Strom et al. have shown that mRNA levels in a biopsy sample can diagnose specific disease states [23,24]. The development of MMDx was based upon the findings of several studies in kidney transplant patients that described and were validated for TCMR, ABMR, acute kidney injury (AKI) and interstitial fibrosis/tubular atrophy (IF/TA) [23,25,26]. In one study, microarray results from 403 kidney transplant biopsies (35 with a diagnosis of TCMR) were used to derive a classifier that assigned TCMR scores to all biopsies; scores were then compared with the histologic diagnosis of the biopsies [25]. Molecular scores correlated with histologic lesions of TCMR (tubulitis and interstitial infiltration) with an accuracy of 89 percent. The positive predictive value (PPV) and negative predictive value (NPV) of a high TCMR molecular score (≥0.1) for predicting the histologic diagnosis of TCMR were 62 and 92 percent, respectively. In a parallel study from the same group, microarray results from the same 403 kidney transplant biopsies (56 with a histologic/donor-specific antibody (DSA) diagnosis of ABMR) were used to derive a classifier that assigned ABMR scores to all biopsies [19,27]. A positive ABMR molecular score (≥0.2) had a PPV and NPV of 64 and 91 percent, respectively, for predicting the histologic/DSA diagnosis of ABMR. In addition, ABMR molecular scores were strongly associated with DSA positivity and were an independent predictor of graft failure. The performance of the TCMR and ABMR molecular scoring systems was prospectively evaluated in a multicenter study of 300 kidney transplant biopsies from 264 patients [28]. Histologic diagnoses of TCMR or ABMR (including C4d-positive ABMR, C4d-negative ABMR and mixed ABMR and TCMR rejection) were present in 11 and 15 percent of biopsies, respectively. A TCMR molecular score of ≥0.1 predicted the histologic diagnosis of TCMR (including borderline rejection) with a PPV and NPV of 49 and 94 percent, respectively. Similarly, an ABMR molecular score of ≥0.2 predicted the diagnosis of ABMR or mixed rejection with PPV and NPV of 50 and 94 percent, respectively. A positive ABMR score in late biopsies (performed more than 1 year post-transplant) was significantly associated with death-censored graft loss at three years (hazard ratio (HR) 2.93, 95% CI 1.97–4.36).

Invasive biomarkers, though crucial in determining an accurate diagnosis, are subject to variability in sampling and interpretation and more often are utilized when the disease process may have reached an irreversible state.

## 3. Non-Invasive Biomarkers

### 3.1. Blood

#### 3.1.1. Donor-Specific Antibodies

The presence of DSAs remains one of the most important biomarkers in the diagnosis of ABMR as defined by the Banff criteria [22]. Many studies have demonstrated that DSAs can not only cause ABMR but are associated with poor long-term allograft survival [29,30]. Though techniques to identify DSAs using Luminex bead assays have increased the sensitivity of their detection, their clinical relevance in predicting onset of ABMR or allograft survival remains unclear [31,32]. Since complement binding of antibodies is a critical step in promoting complement-induced allograft injury, the ability of a DSA to bind to complement has been assessed as a tool to predict their pathogenic significance [33]. Multiple single-center studies in adults and pediatric kidney transplant recipients have shown the association of C3d and C1q binding of DSA to be associated with the risk of ABMR and allograft survival [34,35,36,37]. However, they also show that high MFI value non-complement-fixing DSAs correlate with risk of ABMR and graft loss just as well [38]. Other investigators have not demonstrated the independent association of C3d and C1q fixation with rejection and graft loss [39,40,41]. The intra-graft homing ability of a DSA has also been suggested as a potential marker of its pathogenicity [42,43]. Whether the routine use of studying complement fixation and detecting intra-graft DSA is feasible or will change clinical outcomes remains to be studied further.

#### 3.1.2. Donor-Derived Cell Free DNA

New biomarkers, such as dd-cfDNA, are being developed to overcome the difficulties of detecting the earliest stages of kidney transplant dysfunction and rejection. Although histology obtained via needle biopsy remains the gold standard for diagnosis of rejection, dd-cfDNA serves as an alternative to the invasive renal allograft biopsy and has evolved to a frequently used tool for surveillance and diagnosing of kidney transplant rejection, as well as monitoring of therapy [44,45,46,47]. It is known that histological changes and tissue injury from rejection precedes elevation in serum creatinine by several months [32,48]. During allograft rejection, large amounts of dd-cfDNA are released from the injured allograft into the bloodstream [49]. Methods for measuring dd-cfDNA, including quantitative reverse transcription polymerase chain reaction (PCR) [50], droplet digital PCR [51] and targeted next-generation sequencing [44], were looked at in 102 kidney transplant recipients and correlated with allograft rejection status as determined by renal allograft biopsy [44]. The dd-cfDNA level was able to differentiate between biopsy specimens showing any form of rejection (TCMR or ABMR) and those without rejection. Using a cutoff of 1.0 percent, dd-cfDNA had a PPV and NPV for active rejection of 61 and 84 percent, respectively [44]. Accurate and timely detection of kidney transplant rejection and effective treatment are essential for long-term survival of renal allografts. Up until now, the standard of care was that patients with an elevated plasma dd-cfDNA level commonly underwent a renal allograft biopsy to confirm the presence and type of rejection. There are certain limitations to this biomarker which include patients with multiorgan transplants, pregnant patients and patients with active kidney infection. It is important to point out that dd-cfDNA does not differentiate between TCR and ABMR.

#### 3.1.3. Peripheral Blood Gene Expression Profiling

Routine surveillance (protocol) biopsies have been used in some centers to monitor patients with stable renal function following kidney transplantation, but these are expensive, invasive, pose significant logistical issues and are subject to variability of interpretation, thus limiting wider application. The current standard of care in monitoring patients following kidney transplantation ranges from not using surveillance biopsies at all, using them only in patients at high immunologic risk, to routine use (protocol biopsies) in all patients. Subclinical acute rejection (subAR) is the presence of histological features of acute rejection on renal biopsy in the absence of a decline in renal function. SubAR is present in approximately 25% of surveillance biopsies in renal transplant recipients with stable renal function [52]. Therefore, roughly 75% of surveillance biopsies could be avoided if there was a validated and reliable biomarker test that would distinguish patients with stable renal function who had a quiescent immune profile from those with immune activation.

The TruGraf assay was developed to rule out subclinical rejection in patients with otherwise stable allograft function. Unlike surveillance biopsies which are invasive, it is a DNA microarray-based gene expression blood test that can be obtained easily [53]. The test is based on the analysis of gene-expression “signatures” in peripheral blood that can differentiate a state of immune quiescence, indicating an adequate state of immunosuppression, referred to as transplant excellence (TX), from not-TX, an indication of suboptimal immunosuppression or immune activation. The aim of the TruGraf test is to assist the physician in the assessment of whether the current level of immunosuppression is adequate and to help guide a personalized treatment plan, thereby protecting the function and prolonging graft survival in each individual patient. In the study by Friedewald et al., 192 patients were evaluated across seven sites; the NPV of the test was 91 and 89% in patients with clinical and biopsy-proven phenotypes and in stable patients with biopsy-proven phenotypes, respectively. The PPV of the test was 48% in both groups of the study. The TruGraf test has similar limitations to dd-cfDNA, which include multiorgan transplant recipients, pregnant patients and patients with active kidney infection. How the test performs in patients with graft dysfunction has not been assessed and remains to be studied.

#### 3.1.4. Non-HLA Antibodies

Non-HLA antibodies directed against endothelial receptors play an important role in allograft vascular injury. Lefaucheur et al. showed in a prospective cohort study of 1845 patients that participants with anti-angiotensin II type 1-receptor antibodies (AT1R-Abs)-associated rejection had a higher prevalence of hypertension and more vascular rejection with arterial inflammation [54]. Other studies in adults and pediatric recipients have also shown that AT1R-Abs are associated with an increased risk of allograft loss and a higher incidence of ABMR [54,55,56,57]. Studies across different populations and age groups have determined the cutoff value for AT1R titers in relation to ABMR from 9 to 10 U/mL [56,57,58,59,60]. AT1R-Abs can be considered a non-invasive prognostic tool to enhance the predictive value of histopathology showing features of ABMR to identify patients at risk of accelerated allograft failure.

Like AT1R-Ab, Banasik et al. have shown that anti-ETAR (endothelin 1 type A receptor) antibody is associated with worse graft function at 12 months post-transplant and with more cases of vasculopathy or arteritis in a series of 116 patients [61]. There is growing recognition of the role played by endothelial cell injury in chronic allograft rejection and ABMR. Investigators are looking at anti-endothelial cell antibodies (AECAs) including anti-major histocompatibility complex I antibody (MICA), anti-vimentin, antibodies to endoglin, LG-3 (perlecan), FLT-3, EBIL-3, ICAM-4 and KTR-1 and antibodies to polymorphic proteins [62,63,64,65,66]. Delville et al. developed and performed a unique endothelial cell crossmatch with sera of highly sensitized patients. Using proteomics and transcriptomics they identified new targets of non-HLA antibodies [67]. Undoubtedly, advances in transplant “omics” will help differentiate pathogenic and injurious non-HLA antibodies and develop targeted therapeutics to address the challenging clinical dilemma of non-HLA-mediated ABMR [67].

#### 3.1.5. Peripheral Blood Gene/mRNA Arrays

With the arrival of high throughput sequencing and microarray technologies, the ability to perform molecular assays from peripheral blood has created new possibilities for non-invasive biomarkers in transplantation. The authors of the multicenter prospective BIOMARGIN study identified an eight gene assay with a ROC AUC of 79.9%, to diagnose acute antibody-mediated rejection [68], which has not been validated independently yet.

The authors of the AART study (The Assessment of Acute Rejection in Renal Transplant) developed a 17-gene panel using quantitative PCR from peripheral blood samples called kSORT (Kidney Solid Organ Response Test). kSORT accurately predicted the risk and prevalence of acute rejection with an AUC (area under curve) of 0.92 and a PPV of 93.21% [69]. However, the follow up large retrospective multicenter study conducted to assess the diagnostic performance of kSORT in a real-world setting failed to validate the biomarker [70]. This demonstrates the challenges of implementing biomarkers in the clinical scenario, which requires thorough independent validation.

### 3.2. Urine

Non-invasive and effortless to obtain serially, the urine sample as a source of biomarker has endless potential. The chemokines CXCL9 and CXCL10, when measured in urine, have been shown to serve as a surrogate for non-invasively measuring intra-graft IFN*γ* expression. Rabant et al. showed that combining urinary CXCL10: Cr ratio with detectable DSA significantly improved the accuracy of diagnosing ABMR non-invasively [71]. Urinary CXCL9 and the CXCL9:Cr ratio were mainly associated with tubulointerstitial inflammation, whereas CXCL10 and the CXCL10:Cr ratio were mainly associated with peritubular capillaritis. Of note, though the PPV was low at 25%, urinary CXCL10 and the CXCL10:Cr ratio were strongly associated with ABMR, with > 90% NPV, and may have clinical utility to monitor immunologic quiescence [72].

Utilizing urinary transcriptomics has led to the identification and profiling of distinct mRNA signatures in the urine that are associated with allograft injury [73]. Measurement of mRNA, microRNA and exosomal mRNA in the urine have led to the exciting developments of non-invasive tools to predict allograft rejection [73,74]. Urinary proteomics has also emerged as a potential pathway to discover molecular patterns for detection of ABMR [75].

## 4. Conclusions

The onset of active or chronic ABMR plays a major role in determining the long-term survival of an allograft. It is an exciting era to have a diverse array of predictive and diagnostic biomarkers currently under investigation. Although currently the majority of these tools are not widely available or validated, it is possible that a search for a better diagnostic tool may uncover novel therapeutic options as well. For now, histological examination of the kidney remains the current gold standard for diagnosis of acute rejection, especially in the setting of elevated creatinine. The emergence of a non-invasive, highly sensitive and specific biomarker, or combination of biomarkers, that is independently validated in randomized controlled trials and/or serves as a surrogate for long-term graft survival is eagerly awaited by the transplant community.

## Data Availability

Not applicable.
